# Bias, dispersion, and accuracy of genomic predictions for feedlot and carcase traits in Australian Angus steers

**DOI:** 10.1186/s12711-021-00673-8

**Published:** 2021-09-26

**Authors:** Pâmela A. Alexandre, Yutao Li, Brad C. Hine, Christian J. Duff, Aaron B. Ingham, Laercio R. Porto-Neto, Antonio Reverter

**Affiliations:** 1 CSIRO, Agriculture and Food, Queensland Bioscience Precinct, 306 Carmody Rd., St Lucia, Brisbane, QLD 4067 Australia; 2 CSIRO, Agriculture and Food, F.D. McMaster Laboratory, Chiswick, New England Highway, Armidale, NSW 2350 Australia; 3 Angus Australia, 86 Glen Innes Rd., Armidale, NSW 2350 Australia

## Abstract

**Background:**

Improving feedlot performance, carcase weight and quality is a primary goal of the beef industry worldwide. Here, we used data from 3408 Australian Angus steers from seven years of birth (YOB) cohorts (2011–2017) with a minimal level of sire linkage and that were genotyped for 45,152 SNPs. Phenotypic records included two feedlot and five carcase traits, namely average daily gain (ADG), average daily dry matter intake (DMI), carcase weight (CWT), carcase eye muscle area (EMA), carcase Meat Standard Australia marbling score (MBL), carcase ossification score (OSS) and carcase subcutaneous rib fat depth (RIB). Using a 7-way cross-validation based on YOB cohorts, we tested the quality of genomic predictions using the linear regression (LR) method compared to the traditional method (Pearson’s correlation between the genomic estimated breeding value (GEBV) and its associated adjusted phenotype divided by the square root of heritability); explored the factors, such as heritability, validation cohort, and phenotype that affect estimates of accuracy, bias, and dispersion calculated with the LR method; and suggested a novel interpretation for translating differences in accuracy into phenotypic differences, based on GEBV quartiles (Q1Q4).

**Results:**

Heritability (h^2^) estimates were generally moderate to high (from 0.29 for ADG to 0.53 for CWT). We found a strong correlation (0.73, *P-*value < 0.001) between accuracies using the traditional method and those using the LR method, although the LR method was less affected by random variation within and across years and showed a better ability to discriminate between extreme GEBV quartiles. We confirmed that bias of GEBV was not significantly affected by h^2^, validation cohort or trait. Similarly, validation cohort was not a significant source of variation for any of the GEBV quality metrics. Finally, we observed that the phenotypic differences were larger for higher accuracies.

**Conclusions:**

Our estimates of h^2^ and GEBV quality metrics suggest a potential for accurate genomic selection of Australian Angus for feedlot performance and carcase traits. In addition, the Q1Q4 measure presented here easily translates into possible gains of genomic selection in terms of phenotypic differences and thus provides a more tangible output for commercial beef cattle producers.

**Supplementary Information:**

The online version contains supplementary material available at 10.1186/s12711-021-00673-8.

## Background

Genomic selection represents a revolution in animal breeding. It enables the identification of superior animals through the estimation of genomic estimated breeding values (GEBV) for relevant quantitative traits, and has led to dramatic genetic progress in farm animals during the last two decades [1–3]. This approach is based on the expectation that quantitative trait loci (QTL) are in linkage disequilibrium (LD) with one or more single nucleotide polymorphisms (SNPs) in such a way that a sufficiently dense SNP panel, covering the entire genome, would be able to capture the genetic effects of QTL [4]. Thus, the sum of the estimated effects of all SNP genotypes of an animal is considered to be a predictor of its breeding value [5]. However, the accuracy of GEBV depends on several factors including the size of the reference population, the heritability of the traits and the extent of the LD between SNPs and QTL [6, 7]. One of the most important advantages of genomic selection is the ability to select unproven young candidates; however, accurate predictions are required to support confident decision making. Therefore, Legarra and Reverter [8] have proposed the linear regression (LR) method, which provides population-based semi-parametric estimates of accuracy and bias of GEBV by comparing predictions based on partial and whole data. This cross-validation method has been validated and applied to data from several species, including cattle, sheep, pigs, chickens and trout [9–15]. One recent finding is the need to assess biases and accuracies using various criteria (truncation points) to define partial vs. whole comparisons so that the effect of random variation across years is accounted for [16]. Here, we used the LR method to evaluate GEBV for feedlot and carcase traits in Australian Angus cattle from a dataset spanning seven years of birth cohorts with a minimal level of sire linkage across cohorts.

In beef cattle, genomic prediction offers an opportunity to evaluate, at an early age, traits that are difficult and/or expensive to measure, or can only be measured *post-mortem*, such as carcase traits. Few studies have assessed the predictive accuracies of GEBV for feedlot and carcase traits in cattle. For example, GEBV for average daily weight gain in feedlot-finished Nellore steers that were generated using Bayesian models, have been previously reported with accuracies ranging from 0.18 to 0.27 [17]. Similarly, using Bayesian and genomic BLUP methods, Bolormaa et al. [18] reported an average GEBV accuracy of 0.27 across different carcase and meat quality traits in *Bos taurus*, *Bos indicus*, and composite beef breeds, and a large variation in accuracy between breeds and between traits. Indeed, it is well established that considerable variation exists between breeds for body composition and meat quality traits, further highlighting the importance of evaluating these traits in specific populations [19].

In the Australian cattle herd, Angus is the dominant breed with an estimated 5.6 million females influenced by Angus genetics and accounts for 48% of the national female herd [20]. Considering its importance, our aim was to determine the potential for accurate genomic selection of Australian Angus for feedlot performance, carcase weight and quality by assessing the accuracy of GEBV for these traits using the traditional and the LR methods.

While the LR method has received substantial attention since its development [8], the statistics that it proposes for assessing the quality of genomic predictions have not been widely tested as a function of time (e.g., truncation on year of birth) or with other not time-dependent validation datasets. In addition, changes in GEBV accuracies (and other quality metrics) that are observed due to the use of different models and/or validation populations are usually explored separately for different phenotypes. Further compounding these issues, are the lack of a clear understanding of the relationship between accuracy values and how much extreme individuals based on GEBV will differ in performance. While genetic progress is proportional to accuracy and drives breeding programs for seedstock producers, how changes in accuracy translate to phenotypic differences in commercial settings is poorly understood. An attempt to address this question was reported by [21] in which the distribution of phenotypic values was evaluated after assigning animals to quartiles based on their GEBV.

Here, we complement previous studies in three major aspects: (1) by testing the quality of genomic predictions using the LR method for a complete range of traits that are relevant to feedlot performance and carcase yield and quality and are key components of the beef industry in Australia and worldwide; (2) by exploring the factors, such as heritability estimate, validation cohort and phenotype, that affect the estimates of accuracy, bias and dispersion calculated with the LR method; and (3) by suggesting a novel interpretation for translating differences in accuracy into possible gains of genomic selection in terms of phenotypic differences, providing a more tangible output for beef cattle producers.

## Methods

The data for this study were collected as part of the Australian Angus Sire Benchmarking Program (ASBP), a major initiative of Angus Australia [22] with support from the Meat and Livestock Australia (MLA) and industry partners. This program aims at generating data on steers that were progeny from modern Angus sires, particularly for hard-to-measure traits such as feed efficiency, abattoir carcase measurements, meat quality attributes, and female reproduction. For the development of the ASBP, each cohort of steers included progeny of a genetically diverse range of sires, which were nominated by breeders from all the states of Australia and New Zealand, while some cohorts also included progeny of sires from the USA and the UK. The sires in each cohort were predominantly young bulls (2–3 years of age), with also a few older influential bulls [23]. For the current study, the dataset included phenotypes and fixed effect information for 3408 Australian Angus steers from seven years of birth cohorts (YOB, 2011–2017) for which genotypes for 45,152 autosomal SNPs were available.

The 3408 steers represent 12 breeding properties (herds) and 294 sires with an average of 11.5 progeny per sire, ranging from 1 to 27. In total, 2773 dams were included in the dataset with an average of 1.22 progeny per dam, ranging from 1 to 4. Across the seven YOB cohorts, the numbers of dams with one, two, three, and four progenies were 2221, 485, 65 and 2, respectively. The numbers of progeny (number of sires in brackets) in the YOB cohorts from 2011 to 2017 were 361 (35), 514 (48), 579 (44), 274 (25), 569 (49), 575 (63) and 536 (56), respectively.

Seven phenotypes were analysed including feedlot average daily gain (ADG, kg/d), feedlot average daily dry matter intake (DMI, kg/d), carcase weight (CWT, kg), carcase eye muscle area (EMA, cm^2^), carcase Meat Standards Australia marbling score (MBL, score), carcase ossification score (OSS, score) and carcase subcutaneous rib fat depth (RIB, mm). Table 1 provides summary statistics for these phenotypes. ADG, DMI, CWT, EMA, and RIB were measured as described in [24]. MBL was measured in scores ranging from 100 to 1100 in increments of 10, with higher scores indicating greater marbling [25]. Finally, OSS scores ranged from 100 to 590 in increments of 10, with lower scores indicating less physiological maturity [26, 27].

**Table 1:** Summary statistics including number of records, mean, standard deviation (SD), minimum (Min) and maximum (Max) for traits and covariates included in this study

Category/trait	Number of records	Mean	SD	Min.	Max.
Feedlot					
FAGE, d	3327	511.95	69.39	372.00	767.00
ADG, kg/d	3327	1.59	0.33	0.52	2.90
DMI, kg/d	3327	14.52	2.06	3.36	22.78
Carcase					
CAGE, d	3285	734.53	99.05	504.00	990.00
CWT, kg	3285	432.99	65.60	214.00	607.00
EMA, cm^2^	3273	90.06	10.86	57.00	128.00
MBL	3281	494.66	122.54	160.00	1030.00
OSS	3280	148.25	18.64	100.00	280.00
RIB, mm	3261	17.37	6.04	3.00	41.00

*FAGE* age at feedlot entry, *ADG* average daily gain at feedlot, *DMI* average daily dry matter intake at feedlot, *CAGE* age at carcase assessment, *CWT* carcase weight, *EMA* carcase eye muscle area, *MBL* carcase marbling score, *OSS* carcase ossification score, *RIB* carcase subcutaneous fat depth measured between the 5th and 13th rib

Variance components, heritabilities, and genetic and residual correlations were estimated using the Qxpak5 software package [28]. For this purpose, a linear mixed model was used to analyse all traits, which included a fixed effect of contemporary group (CG), i.e. an amalgamation of property of origin, year and month of birth, and date of measurement, and effects of age of dam (AOD) at birth of the calf (in years) and age at measurement (as a linear covariate). CG were not the same for feedlot and carcase traits because measurement dates differed. In addition, the random additive polygenic and residual effects were fitted with assumed distributions $N(\mathbf{0}, \mathbf{G}\otimes{\mathbf{V}}_{\mathbf{G}})$ and $N(\mathbf{0}, \mathbf{I}\otimes{\mathbf{V}}_{\mathbf{R}})$, respectively, where $\mathbf{G}$ represents the genomic relationship matrix (GRM) generated using the first method of VanRaden [29], ${\mathbf{V}}_{\mathbf{G}}$ is the genetic variance–covariance matrix, $\mathbf{I}$ is an identity matrix, ${\mathbf{V}}_{\mathbf{R}}$ is the residual covariance matrix and $\otimes$ represents the Kronecker product. Two different analyses were undertaken to generate estimates for the *whole* and *partial* datasets. First, a multivariate (7-variate) analysis was performed with all seven traits. The resulting GEBV from this multivariate analysis are termed ${\widehat{\mathbf{u}}}_{\mathrm{w}}$ to indicate that they are based on the *whole* dataset and will be used as the calibration in the computation of the accuracy and bias with the LR method. Next, a series of 49 univariate analyses were undertaken each with a single trait and where the values for animals from consecutive YOB cohorts were treated as missing. Hence, 49 analyses were performed originating from seven traits by seven YOB cohorts. The resulting GEBV from these univariate analyses are termed ${\widehat{\mathbf{u}}}_{p}$ to indicate that they are based on *partial* data and will be used as validation data.

To ascertain the quality of the resulting GEBV in the validation population (i.e. the elements of ${\widehat{\mathbf{u}}}_{p}$ corresponding to the focal individuals in the validation population), we used the following four metrics:

(1) Traditional accuracy (${\mathrm{ACC}}_{\mathrm{T}}$): Pearson’s correlation ($r$) between a GEBV and its associated adjusted phenotype (${\mathbf{y}}^{*}$; phenotype $y$ adjusted for CG fixed effects and covariates) for individuals in the validation population was divided by the square root of the heritability [18]:

$${\text{ACC}}_{{\text{T}}} = \frac{{r\left( {{\hat{\mathbf{u}}}_{p} - {\mathbf{y}}^{*} } \right)}}{{\sqrt {h^{2} } }}.$$

(2) Bias calculated with the LR method (${\text{Bias}}_{{{\text{LR}}}} )$: is the difference between the average GEBV of individuals in the validation population using the partial data minus that using the whole data [8, 15]:

$${\text{Bias}}_{{{\text{LR}}}} = \overline{{{\hat{\mathbf{u}}}_{p} }} - \overline{{{\hat{\mathbf{u}}}_{w} }} .$$

(3) Dispersion calculated with the LR method (${\text{Disp}}_{{{\text{LR}}}}$): for individuals in the validation population, dispersion was measured from the slope of the regression of ${\hat{\mathbf{u}}}_{w}$ on ${\hat{\mathbf{u}}}_{p}$ [8, 15]:

$${\text{Disp}}_{{{\text{LR}}}} = \frac{{cov\left( {{\hat{\mathbf{u}}}_{w} ,\user2{ }{\hat{\mathbf{u}}}_{{\varvec{p}}} } \right)}}{{var\left( {{\hat{\mathbf{u}}}_{p} } \right)}}.$$

(4) Accuracy calculated with the LR method (${\text{ACC}}_{{{\text{LR}}}} )$: for individuals in the validation population, ${\text{ACC}}_{{{\text{LR}}}}$ was computed as follows [8, 15]:

$${\text{ACC}}_{{{\text{LR}}}} = \sqrt {\frac{{cov\left( {{\hat{\mathbf{u}}}_{w} ,\user2{ }{\hat{\mathbf{u}}}_{{\varvec{p}}} } \right)}}{{\left( {1 + \overline{F} - 2\overline{f}} \right)\sigma_{g, \infty }^{2} }}} ,$$

where $\overline{F}$ is the average inbreeding coefficient, $2\overline{f}$ is the average relationship between individuals, and $\sigma_{g, \infty }^{2}$ is the genetic variance at equilibrium in a population under selection. Assuming the individuals in the validation population are not under selection, $\sigma_{g, \infty }^{2}$ was estimated by the additive genetic variance estimated from the partial dataset.

Then, to characterise the factors affecting the GEBV quality metrics, accuracy, bias and dispersion were treated as dependent variables in an ANOVA model that included h^2^ estimate, validation cohort and trait as independent predictor variables.

Finally, using only the animals in the validation population, we ranked animals based on GEBV, identified those in the highest (Q1) and lowest (Q4) quartiles of the GEBV scale, and calculated the difference (Q1Q4) between the adjusted phenotypes of these two sets of animals. Then, we used the following models to evaluate the relationship between individual GEBV accuracy metrics and Q1Q4 using the PROC GLM program (SAS Inst. Inc.):

$${\text{Q}}1{\text{Q}}4 = {\text{Trait}} + {\text{Cohort}} + {\text{ACC}}_{{\text{T}}} + {\mathbf{e}},$$

$${\text{Q}}1{\text{Q}}4 = {\text{Trait}} + {\text{Cohort}} + {\text{ACC}}_{{{\text{LR}}}} + {\mathbf{e}},$$

where ${\text{Q}}1{\text{Q}}4$ is the difference, in SD units, between the highest and the lowest quartile for adjusted phenotypes based on GEBV ranking, ${\text{Trait}}$ corresponds to the seven phenotypes analysed, ${\text{Cohort}}$ corresponds to the seven validation cohorts, and ${\mathbf{e}}$ is the vector of residual effects.

## Results

In this study, we used data from 3408 Australian Angus steers from seven YOB cohorts (2011 to 2017). These steers represented 294 sires from 12 breeding properties (or herds). A low level of sire linkage across cohorts was identified (see Additional file 1 Table S1) as was intended in the ASBP design. The 12 breeding properties contributed on average 284 animals ranging from 57 to 495 and all except two contributed animals across three YOB cohorts. One breeding property was represented in a single YOB cohort while another one was represented in five YOB cohorts (see Additional file 1: Table S2). These sire and breeding property linkages across YOB cohorts can have an impact on the accuracies of GEBV since each cohort is used as the validation population. Of note, the GRM showed that the within- (i.e. diagonals of the GRM) and between-animal relationships (off-diagonals of the GRM) were close to the expected values of 1 and 0, respectively (see Additional file 1 Table S3). Equally interesting, was the very similar variation that we observed across these two types of relationships, which indicates a single population from the point of view of genetic variation [30].

Heritability estimates were generally moderate to high, ranging from 0.30 for ADG to 0.53 for CWT (Table 2). Genetic correlations were strong and positive between ADG and DMI (0.59) and between ADG and CWT (0.65) and close to zero between MBL and OSS (− 0.01) and between MBL and RIB (− 0.09). In general, the estimates of the residual correlation were lower and closer in magnitude to zero than the genetic correlations. For instance, between the growth traits ADG, DMI and CWT, the genetic and residual correlations were estimated at ~ 0.60 and ~ 0.30, respectively. Finally, except for CWT, the estimates of the genetic and residual correlations between feedlot and carcase traits were weak.

**Table 2:** Genomic estimates of heritability (italics on the diagonal), genetic (above the diagonal) and residual (below the diagonal) correlations for feedlot and carcase traits

	ADG	DMI	CWT	EMA	MBL	OSS	RIB
ADG	*0.30*	0.59	0.65	0.15	0.05	0.08	0.11
DMI	0.31	*0.38*	0.63	0.12	0.10	0.10	0.16
CWT	0.25	0.38	*0.53*	0.37	0.04	0.13	0.18
EMA	0.13	0.15	0.48	*0.45*	0.14	0.03	− 0.17
MBL	0.01	0.04	0.08	0.18	*0.42*	− 0.01	− 0.09
OSS	− 0.04	0.01	0.05	0.05	0.02	*0.33*	0.00
RIB	0.02	0.06	0.19	− 0.01	-0.03	0.07	*0.31*

*ADG* average daily gain at feedlot, *DMI* average daily dry matter intake at feedlot, *CWT* carcase weight, *EMA* carcase eye muscle area, *MBL* carcase marbling score, *OSS* carcase ossification score, *RIB* carcase subcutaneous fat depth at the ribs level

**Fig. 1:**
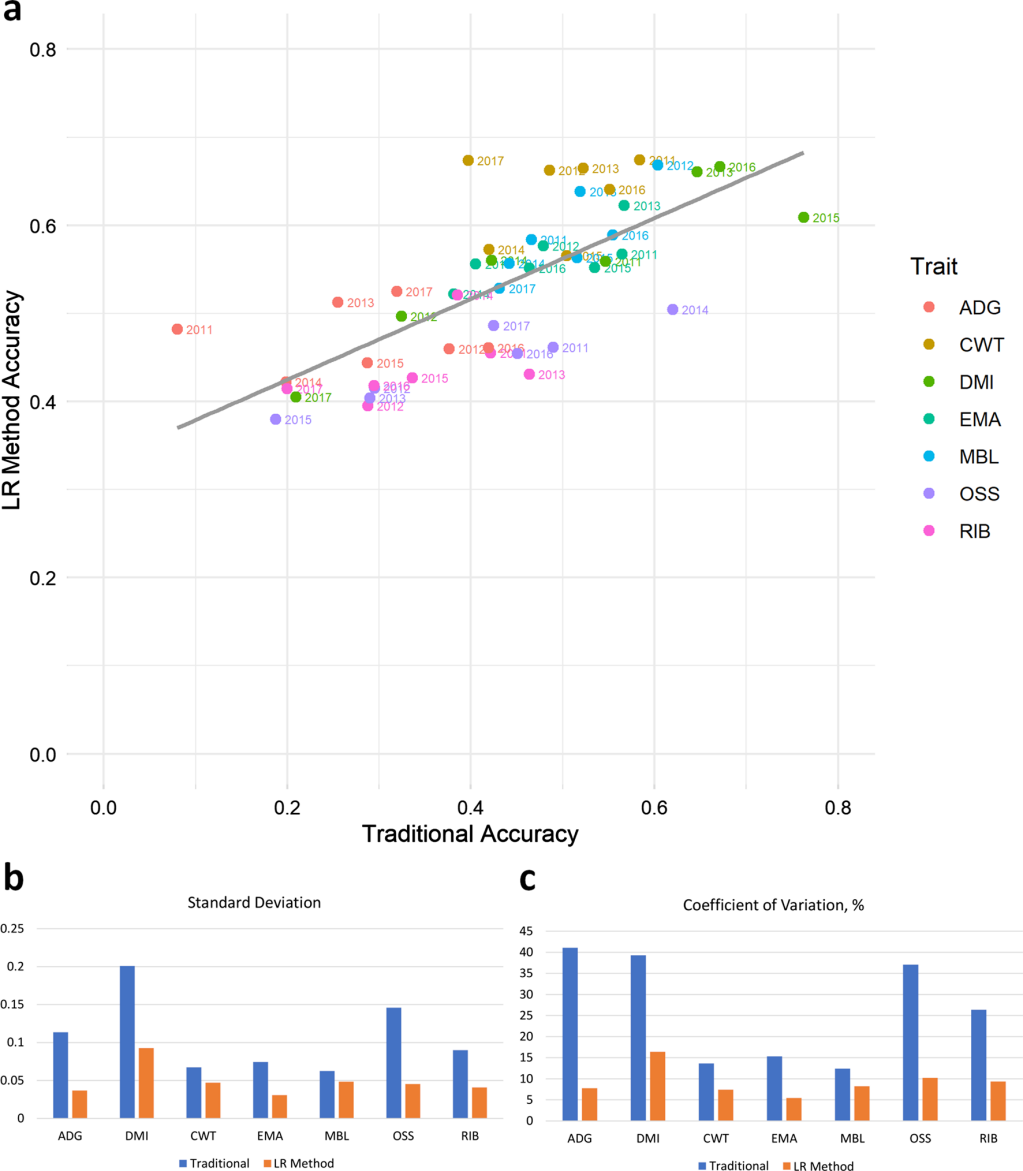
Relationship between traditional accuracies and accuracies obtained with the LR method considering seven years of birth cohorts (2011 to 2017) and the seven traits analysed: average daily gain at feedlot (ADG), average daily dry matter intake at feedlot (DMI), carcase weight (CWT), carcase eye muscle area (EMA), carcase marbling score (MBL), carcase ossification score (OSS), carcase subcutaneous fat depth at the ribs level (RIB). **a** Scatter plot across the 49 accuracy values (i.e. seven traits by seven cohorts); **b** within trait, across year standard deviation; and **c** within trait, across year coefficient of variation

The four GEBV quality metrics (ACC_T_, ACC_LR_, Bias_LR_, and Disp_LR_, see in “Methods” section) are in Table 3. ACC_T_ ranged from 0.28 for ADG to 0.51 for DMI, while ACC_LR_ ranged from 0.44 for RIB to 0.64 for CWT. We found a strong correlation of 0.73 (*P*-value < 0.001) between ACC_T_ and ACC_LR_ (Fig. 1a). ACC_LR_ were on average lower than ACC_T_ (Table 3) and more variable (Fig. 1b). This resulted in a much higher coefficient of variation for ACC_LR_ (Fig. 1c), particularly for ADG (41.06 vs. 7.79%) and OSS (37.07 vs. 10.24%). For all the traits, the Bias_LR_ values were close to 0 and the Disp_LR_ values close to 1 (Table 3), as expected in the absence of bias.

**Table 3:** Traditional (ACC_T_) and LR (ACC_LR_) accuracies, LR bias (Bias_LR_) and LR dispersion (Disp_LR_) of GEBV for feedlot and carcase traits from a 7-way cross-validation^a^ scheme based on year of birth (YOB) cohorts

	ADG	DMI	CWT	EMA	MBL	OSS	RIB
ACC_T_							
Mean	0.28	0.51	0.49	0.48	0.50	0.39	0.34
SD	0.11	0.20	0.07	0.07	0.06	0.15	0.09
Min.	0.08	0.21	0.40	0.38	0.43	0.19	0.20
Max.	0.42	0.76	0.58	0.57	0.60	0.62	0.46
ACC_LR_							
Mean	0.47	0.57	0.64	0.56	0.59	0.44	0.44
SD	0.04	0.09	0.05	0.03	0.05	0.05	0.04
Min.	0.42	0.41	0.57	0.52	0.53	0.38	0.40
Max.	0.53	0.67	0.67	0.62	0.67	0.50	0.52
Bias_LR_							
Mean	0.00	0.03	0.27	− 0.03	− 0.08	− 0.07	0.02
SD	0.01	0.04	0.61	0.16	1.71	0.16	0.07
Min.	− 0.01	− 0.01	− 0.54	− 0.18	− 2.14	− 0.25	− 0.05
Max.	0.01	0.08	1.20	0.31	2.13	0.22	0.14
Disp_LR_							
Mean	0.97	1.12	0.99	0.94	0.98	0.93	0.93
SD	0.15	0.49	0.09	0.12	0.09	0.10	0.13
Min.	0.74	0.37	0.83	0.78	0.88	0.76	0.72
Max.	1.17	1.68	1.10	1.13	1.13	1.05	1.12

^a^Refer to Tables S4 to 7 [see Additional file 1: Tables S4–S7] for the individual results on a per cohort basis

*ADG* average daily gain at feedlot, *DMI* average daily dry matter intake at feedlot, *CWT* carcase weight, *EMA* carcase eye muscle area, *MBL* carcase marbling score, *OSS* carcase ossification score, *RIB* carcase subcutaneous fat depth at the ribs level

In magnitude, the Bias_LR_ for CWT (on average 0.27 kg; Table 3) appears to be larger than that observed for the other traits. However, in relative terms, this bias is equivalent for all traits. For instance, the SD of the GEBV Bias_LR_ is 0.61 kg and 0.16 cm^2^ for CWT and EMA, respectively (Table 3), which are equal to ~ 1% of the SD observed for each trait (Table 1).

By investigating the effects of heritability, validation cohort and trait on the GEBV quality metrics (Table 4) we found that, in the cross-validation scheme and for a given trait, there is a significant negative correlation between the estimated heritability and the slope of the dispersion (r = − 0.56 ± 0.089; *P*-value < 0.001). Based on the coefficient of determination (R^2^), a model that includes the effects of heritability estimate, validation cohort and trait explained 65.3, 84.9, 14.5 and 73.3% of the variation in ACC_T_, ACC_LR_, Bias_LR_ and Disp_LR_, respectively. Thus, validation cohort, trait, or heritability of the trait did not significantly affect the Bias_LR_ of GEBV. In addition, it is important to note that validation cohort was not a significant source of variation (*P*-value > 0.10) for any of the four GEBV quality metrics (Table 4).

**Table 4:** P-value and coefficient of determination (R^2^) of the effect of heritability (h^2^), validation cohort and trait on GEBV quality metrics, including traditional accuracy (ACC_T_) and accuracy (ACC_LR_), bias (Bias_LR_) and dispersion (Disp_LR_) obtained with the LR method

	ACC_T_	ACC_LR_	Bias_LR_	Disp_LR_
h^2^	0.0028	0.0001	0.6187	0.0001
Cohort	0.1514	0.2805	0.5862	0.1176
Trait	0.0001	0.0001	0.0714	0.0001
R^2^, %	65.3	84.9	14.5	73.3

After ranking the validation animals according to their GEBV and calculating the phenotypic differences (Q1Q4) between animals in the highest and lowest GEBV quartile (Table 5), we observed that, averaged across the 49 estimates (7 cohorts and 7 traits), the estimate of the Q1Q4 difference is 5.59-fold larger than its SE, which indicates the consistency of this metric. When expressed in SD units (Table 5, last row), the smallest (0.35) and largest (0.94) Q1Q4 differences were found for ADG and CWT, respectively. After adjusting for the effects of trait (*P-*value < 0.0001) and validation cohort (*P-*value > 0.05), we found that for each 0.1 increase in ACC_LR_, the Q1Q4 difference increased by an average of 0.132 SD units, while for each 0.1 increase in ACC_T_ this increase was smaller, i.e. 0.081 SD units. In both cases, the intercept did not significantly differ from zero (*P-*value > 0.05).

**Table 5:** Average difference (± SE) in adjusted phenotypes between the highest and lowest quartile (Q1Q4) based on GEBV ranking

Cohort	ADG	DMI	CWT	EMA	MBL	OSS	RIB
2011	0.00 ± 0.04	1.05 ± 0.17	33.57 ± 4.54	7.70 ± 1.16	103.47 ± 16.42	10.78 ± 1.87	3.56 ± 0.83
2012	0.14 ± 0.03	0.85 ± 0.21	33.25 ± 4.17	7.49 ± 1.05	116.36 ± 13.93	7.47 ± 1.92	2.21 ± 0.57
2013	0.08 ± 0.03	1.27 ± 0.17	34.44 ± 3.86	9.45 ± 1.06	99.20 ± 11.69	5.94 ± 1.62	3.35 ± 0.54
2014	0.10 ± 0.04	1.21 ± 0.23	25.90 ± 5.37	4.70 ± 1.39	78.60 ± 16.43	10.34 ± 1.72	3.78 ± 0.94
2015	0.08 ± 0.03	1.29 ± 0.17	28.36 ± 3.32	7.12 ± 0.95	85.45 ± 10.64	5.31 ± 1.57	2.13 ± 0.49
2016	0.13 ± 0.03	1.31 ± 0.19	31.51 ± 3.31	6.49 ± 0.98	86.56 ± 10.60	9.30 ± 1.49	1.78 ± 0.50
2017	0.09 ± 0.03	0.85 ± 0.19	20.53 ± 3.24	5.13 ± 0.94	60.19 ± 9.88	8.48 ± 1.55	0.96 ± 0.43
Average	0.09 ± 0.03	1.12 ± 0.19	29.65 ± 3.97	6.87 ± 1.07	89.98 ± 12.79	8.23 ± 1.68	2.54 ± 0.61
Average/SD*	0.35	0.71	0.94	0.83	0.89	0.61	0.55

^*^Standard deviation of adjusted phenotypes

## Discussion

Genomic predictions need to be accurate to be successfully implemented. The accuracy of predictions depends highly on size of the reference population, relatedness between test animals and those in the reference population, and heritability of the target traits, but it can also vary between different breeds and populations [18]. Here, we tested the accuracy of genomic predictions for seven feedlot and carcase traits that were generated using 3408 Australian Angus steers genotyped for 45,152 SNPs. Our estimates of genetic parameters for Australian Angus were all genomic-based and no pedigree data was used in the estimation process. Heritability estimates as well as ACC_T_ and ACC_LR_ were moderate to high. ACC_T_ were highly correlated with ACC_LR_. Since the lowest ACC_LR_ value obtained was 0.44 (for RIB and OSS), and the measures of bias and dispersion fell within expected values, our results provide evidence of the potential for accurate genomic selection of the evaluated traits in Australian Angus cattle.

We have shown that the 7-way cross-validation scheme implemented here, based on YOB cohorts within the same population, is as accurate as genomic prediction using a training set from a different (target) subpopulation [9]. In that work [9], the authors argued that genomic predictions using genetically heterogeneous training sets could provide more flexibility and showed that a training set that includes animals from genetically related lines can be as valuable as a training set from the target population. In our study, since the YOB cohorts used to generate the validation populations presented a low level of sire linkage, we could use this experimental design.

Heritability estimates ranged from 0.30 for ADG to 0.53 for CWT which is consistent with previously reported values. For instance, Somavilla et al. [17] using Bayesian genomic best linear unbiased prediction (GBLUP) to evaluate feedlot ADG in Nellore cattle reported a heritability of 0.31, and Su et al. [31] reported heritabilities of 0.48 and 0.43 for marbling score and of 0.51 and 0.34 for CWT, in Hereford and Simmental cattle, respectively. In Angus cattle, a previous study using animals from the ASBP but based on pedigree information only, reported heritabilities of 0.33, 0.34, 0.52, 0.55 and 0.66 for ADG, RIB, EMA, DMI, and CWT, respectively [24].

Genetic correlations were high and positive between feedlot and weight traits (ADG, DMI and CWT) and close to zero between carcase quality traits (MBL, OSS and RIB). Moreover, low correlations were observed between these two groups of traits. These results corroborate the findings from previous studies that found lower correlations between live/carcass weight and traits such as fat deposition and marbling [32]. Particularly in Angus cattle, similar results based on pedigree information have been reported using a subset of six [24] and four [33] of the seven YOB cohorts used here. In those studies, the standard error (SE) associated with pedigree-based estimates of h^2^ and genetic correlation ranged from 0.06 to 0.11 and from 0.04 to 0.27, respectively. In the literature on livestock genomics, there is ample evidence showing that the SE associated with genomic estimates of genetic parameters is lower than that associated with pedigree-based estimates (see for instance [34–36]), which is attributed to the genomic relationship matrix being more informative than the pedigree-based numerator relationship matrix.

Based on a simulation study, Macedo et al. [15] showed that the LR method works in the presence of selection and verified that LR accuracies agreed with theoretical accuracies once the Bulmer effect is correctly accounted for. In the current study, we used real data and report that the ACC_T_ and ACC_LR_ for each trait were highly correlated (r = 0.73; *P*-value < 0.001). One key advantage of the LR method for computing accuracy is that it does not need adjustment factors to pre-correct phenotypes, which are themselves estimates and prone to errors, for instance, in situations with many contemporary groups each with few records or when heritability is poorly estimated (i.e. when the selection process is inadequately described in the data and environmental trends are present). Instead, the LR method obviates the need for adjustment factors and has been shown to perform optimally even if the model uses an incorrect heritability or if a hidden trend exists in the data [15].

It is worth noting that the complete dataset was used to obtain estimates of CG fixed effects and covariates, and these estimates were used to adjust the phenotypes of individuals in the validation population. These adjusted phenotypes were needed in the computation of ACC_T_ and Q1Q4. Animals in the validation and training sets were raised in different CG. Therefore, the only linkage between these animals is through genomic relationships and no link was created as a consequence of using records in the validation sets to obtain the estimates for the precorrection. However, while the key advantage of ACC_LR_ is that is does not require to estimate adjustment factors from fixed effects corresponding to the validation population, whether that is a sufficient argument to favour ACC_LR_ over ACC_T_ cannot be determined with certainty because it is likely that they are capturing different aspects of predictions.

In agreement with previous studies, our results suggest that the accuracy for carcase traits is higher than for live animal body composition traits [37] and that the accuracy is higher for traits with a higher heritability [18, 38]. In fact, a high correlation (r = 0.91, *P-*value < 0.001) was observed between heritability and GEBV accuracy. An absence of GEBV bias was indicated by values close to zero for all traits. Bias was not significantly influenced by validation cohort, heritability of the trait, or trait. In the absence of bias, the expected value of dispersion is 1. Although a negative correlation between heritability and dispersion was observed, such that higher estimates of heritability were associated with overdispersion in the resulting GEBV, Disp_LR_ values were mostly around 1, ranging from 0.93 for OSS and RIB to 1.12 for DMI.

The breeding properties that contributed data to the ASBP were selected on a YOB basis and on their ability to supply data on hard-to-measure traits and from sires that were not already represented in other YOB. This particular structure allows for a unique paradigm by which each YOB cohort can be considered as a truly independent validation dataset to generate the “partial” GEBV which, in turn, gives us the opportunity to better test the optimality of the genomic predictions than if the partial datasets were generated at random or based on the last generation (as often used to mimic the “old” versus the “recent” predictions). Indeed, analysis of the variability of accuracy estimates within and across traits and years revealed that ACC_LR_ were less affected by random variation within trait across years (Figs. 1b and c) and within year across traits than ACC_T_. Averaged across the seven YOB cohorts, the SD of ACC_LR_ was 0.09 compared to 0.14 for ACC_T_.

To further characterise the factors that affect GEBV quality metrics, accuracy, bias, and dispersion were treated as dependent variables in an ANOVA model that included h^2^ estimate, validation cohort and trait as independent predictor variables (Table 4). We confirmed that bias was not significantly affected by any of the independent variables (*P*-value > 0.05). Similarly, in spite of the low level of sire linkage across cohorts and the varying size of the cohorts (274–579), validation cohort was not a significant source of variation for any of the GEBV quality metrics.

The high correlation between heritability and GEBV accuracy was also reflected in the phenotypic differences between validation animals in the highest and lowest GEBV quartile (Q1Q4). The higher was the GEBV accuracy, the larger was the phenotypic difference between quartiles and, therefore, the greater was the genetic gain which could be expected when selecting for the trait. Moreover, we found a larger increase in Q1Q4 difference (0.132 SD units) for each 0.1 increase in ACC_LR_ than that (0.081 SD units) for the same 0.1 increase in ACC_T_. These results suggest an improved ability of ACC_LR_ to discriminate between extreme GEBV quartiles. The fact that both intercepts were not significantly different from zero indicates that when either ACC_T_ or ACC_LR_ is zero, GEBV are not different from randomly guessed values, and hence, the Q1Q4 difference is zero, as expected.

## Conclusions

We have performed a series of analyses aimed at investigating the behaviour of bias, dispersion, and accuracy of GEBV according to the characteristics of the validation dataset, and the value of these quality metrics for reflecting extreme-performing individuals. The GEBV quality metrics based on the LR method, i.e. accuracy, bias, and dispersion, as well as the heritabilities reported here, suggest that there is potential for accurate genomic selection of Australian Angus for feedlot performance and carcase weight and quality.

## Supplementary Information

**Additional file 1: Table S1**. Levels of sire linkage (negligible) across cohorts. The data provided represent the level of sire linkage across the year of birth cohorts used as validation population. **Table S2**. Breeding property linkages across cohorts. The data provided represent the breeding property linkages across the year of birth cohorts used as validation population. **Table S3**. Summary statistics for the genomic relationship matrix (GRM) values computed using Method 1 of VanRaden [29]. The data provided represent the summary statistics for the genomic relationship matrix (GRM) values computed using Method 1 of VanRaden [29]. **Table S4**. Accuracy (ACCT) of GEBV for feedlot and carcase traits from a 7-way cross-validation scheme based on YOB cohorts. The data provided represent the accuracy (computed from the correlation between GEBV and adjusted phenotypes in the validation population divided by the square root of heritability) of GEBV for feedlot and carcase traits from a 7-way cross-validation scheme based on year of birth cohorts used as validation population. **Table S5**. Estimates of GEBV bias using the LR method (BiasLR) for feedlot and carcase traits from a 7-way cross-validation scheme based on YOB cohorts. The data provided represent the estimates of GEBV bias using the LR method for feedlot and carcase traits from a 7-way cross-validation scheme based on year of birth cohorts used as validation population. **Table S6**. Estimates of GEBV dispersion using the LR method for feedlot and carcase traits from a 7-way cross-validation scheme based on YOB cohorts. The data provided represent the estimates of GEBV dispersion using the LR method (DispLR) for feedlot and carcase traits from a 7-way cross-validation scheme based on year of birth cohorts used as validation population. **Table S7**. Estimates of GEBV accuracy using the LR method (ACCLR) for feedlot and carcase traits from a 7-way cross-validation scheme based on YOB cohorts. The data provided represent the estimates of GEBV accuracy using the LR method for feedlot and carcase traits from a 7-way cross-validation scheme based on year of birth cohorts used as validation population.

## Data Availability

The datasets used and analysed during the current study are available from the corresponding author on reasonable request and upon signing a data transfer agreement.
