# Correction to: Bias, dispersion, and accuracy of genomic predictions for feedlot and carcase traits in Australian Angus steers

**DOI:** 10.1186/s12711-021-00677-4

**Published:** 2021-11-15

**Authors:** Pâmela A. Alexandre, Yutao Li, Brad C. Hine, Christian J. Duff, Aaron B. Ingham, Laercio R. Porto-Neto, Antonio Reverter

**Affiliations:** 1 CSIRO, Agriculture and Food, Queensland Bioscience Precinct, 306 Carmody Rd., St Lucia, Brisbane, QLD 4067 Australia; 2 CSIRO, Agriculture and Food, F.D. McMaster Laboratory, Chiswick, New England Highway, Armidale, NSW 2350 Australia; 3 Angus Australia, 86 Glen Innes Rd., Armidale, NSW 2350 Australia

## Correction to: Genet Sel Evol (2021) 53: 77 10.1186/s12711-021-00673-8

After the publication of the original article [1], the authors noticed that there was an error.

In the first paragraph of page 5, where we compare ACC_LR_ and ACC_T_, the accuracy symbols have been swapped. The right sentences are: “**ACC**_**T**_ were on average lower than **ACC**_**LR**_ (Table 3) and more variable (Fig. 1b). This resulted in a much higher coefficient of variation for **ACC**_**T**_ (Fig. 1c), particularly for ADG (41.06 vs. 7.79%) and OSS (37.07 vs. 10.24%).”

The related Table 3, Fig. 1 and the discussion around those results are correct.
