# Supplementary material for: Bias, dispersion, and accuracy of genomic predictions for feedlot and carcase traits in Australian Angus steers
Source: Genet Sel Evol. 2021 Sep 26;53:77. doi: 10.1186/s12711-021-00673-8 (PMC8474816; doi:10.1186/s12711-021-00673-8)
Supplement: Supplementary file 1 — Additional file 1: Table S1. Levels of sire linkage (negligible) across cohorts. The data provided represent the level of sire linkage across the year of birth cohorts used as validation population. Table S2. Breeding property linkages across cohorts. The data provided represent the breeding property linkages across the year of birth cohorts used as validation population. Table S3. Summary statistics for the genomic relationship matrix (GRM) values computed using Method 1 of VanRaden [29]. The data provided represent the summary statistics for the genomic relationship matrix (GRM) values computed using Method 1 of VanRaden [29]. Table S4. Accuracy (ACCT) of GEBV for feedlot and carcase traits from a 7-way cross-validation scheme based on YOB cohorts. The data provided represent the accuracy (computed from the correlation between GEBV and adjusted phenotypes in the validation population divided by the square root of heritability) of GEBV for feedlot and carcase traits from a 7-way cross-validation scheme based on year of birth cohorts used as validation population. Table S5. Estimates of GEBV bias using the LR method (BiasLR) for feedlot and carcase traits from a 7-way cross-validation scheme based on YOB cohorts. The data provided represent the estimates of GEBV bias using the LR method for feedlot and carcase traits from a 7-way cross-validation scheme based on year of birth cohorts used as validation population. Table S6. Estimates of GEBV dispersion using the LR method for feedlot and carcase traits from a 7-way cross-validation scheme based on YOB cohorts. The data provided represent the estimates of GEBV dispersion using the LR method (DispLR) for feedlot and carcase traits from a 7-way cross-validation scheme based on year of birth cohorts used as validation population. Table S7. Estimates of GEBV accuracy using the LR method (ACCLR) for feedlot and carcase traits from a 7-way cross-validation scheme based on YOB cohorts. The data provided represent the estima [file 12711_2021_673_MOESM1_ESM.docx]

**Supplementary Tables**

**Supplementary Table 1.** Sire linkages (negligible) across cohorts.

| Cohort^1^ | 2011 | 2012 | 2013 | 2014 | 2015 | 2016 | 2017 |
| --- | --- | --- | --- | --- | --- | --- | --- |
|  |  |  |  |  |  |  |  |
| 2011 | 35 | 1 | 0 | 0 | 0 | 0 | 0 |
| 2012 | 1 | 48 | 4 | 0 | 0 | 0 | 0 |
| 2013 | 0 | 4 | 44 | 4 | 1 | 0 | 0 |
| 2014 | 0 | 0 | 4 | 25 | 0 | 0 | 0 |
| 2015 | 0 | 0 | 1 | 0 | 49 | 1 | 1 |
| 2016 | 0 | 0 | 0 | 0 | 1 | 63 | 14 |
| 2017 | 0 | 0 | 0 | 0 | 1 | 14 | 56 |

^1^Year of birth cohort used as the validation population.

**Supplementary Table 2.** Breeding property linkages across cohorts.

| Cohort^1^ | 2011 | 2012 | 2013 | 2014 | 2015 | 2016 | 2017 |
| --- | --- | --- | --- | --- | --- | --- | --- |
|  |  |  |  |  |  |  |  |
| 2011 | 5 | 5 | 5 | 1 | 1 | 0 | 0 |
| 2012 | 5 | 5 | 5 | 1 | 1 | 0 | 0 |
| 2013 | 5 | 5 | 5 | 1 | 1 | 0 | 0 |
| 2014 | 1 | 1 | 1 | 3 | 3 | 2 | 0 |
| 2015 | 1 | 1 | 1 | 3 | 7 | 6 | 4 |
| 2016 | 0 | 0 | 0 | 2 | 6 | 6 | 4 |
| 2017 | 0 | 0 | 0 | 0 | 4 | 4 | 5 |

^1^Year of birth cohort used as the validation population.

**Supplementary Table 3.** Summary statistics for the genomic relationship matrix (GRM) values computed using Method 1 of VanRaden (2008).

| Element | N | Mean | SD | Min. | Max. |
| --- | --- | --- | --- | --- | --- |
|  |  |  |  |  |  |
| Diagonal | 3,408 | 0.9959 | 0.0269 | 0.9180 | 1.1408 |
| Off-Diagonal | 5,805,528 | -0.0003 | 0.0274 | -0.1022 | 0.9678 |

**Supplementary Table 4**. Accuracy^1^ (ACC_T_) of GEBV for feedlot and carcase traits from a 7-way cross-validation schema based on YOB cohorts

| Cohort^2^ | ADG | DMI | CWT | EMA | MSA | OSS | RIB | Average |
| --- | --- | --- | --- | --- | --- | --- | --- | --- |
| 2011 | 0.0800 | 0.5467 | 0.5839 | 0.5644 | 0.4655 | 0.4892 | 0.4215 | 0.4502 |
| 2012 | 0.3762 | 0.3241 | 0.4856 | 0.4788 | 0.6034 | 0.2950 | 0.2873 | 0.4072 |
| 2013 | 0.2550 | 0.6462 | 0.5222 | 0.5669 | 0.5189 | 0.2895 | 0.4633 | 0.4660 |
| 2014 | 0.1985 | 0.4224 | 0.4193 | 0.3811 | 0.4413 | 0.6198 | 0.3851 | 0.4097 |
| 2015 | 0.2871 | 0.7622 | 0.5039 | 0.5347 | 0.5152 | 0.1869 | 0.3359 | 0.4465 |
| 2016 | 0.4189 | 0.6709 | 0.5509 | 0.4636 | 0.5541 | 0.4504 | 0.2944 | 0.4862 |
| 2017 | 0.3190 | 0.2089 | 0.3968 | 0.4050 | 0.4310 | 0.4247 | 0.1994 | 0.3407 |
| Average | 0.2764 | 0.5116 | 0.4946 | 0.4849 | 0.5042 | 0.3936 | 0.3410 |  |

^1^Traditional accuracy computed from the correlation between GEBV and adjusted phenotypes in the validation population divided by the square root of heritability.

^2^Year of birth cohort used as the validation population.

ADG = average daily gain at feedlot; DMI = average daily dry matter intake at feedlot; CWT = carcase weight; EMA = carcase eye muscle area; MBL = carcase marbling score; OSS = carcase ossification score; RIB = carcase subcutaneous fat depth at the ribs level.

**Supplementary Table 5**. LR Method estimates of GEBV bias (Bias_LR_) for feedlot and carcase traits from a 7-way cross-validation schema based on YOB cohorts

| Cohort^1^ | ADG | DMI | CWT | EMA | MSA | OSS | RIB | Average |
| --- | --- | --- | --- | --- | --- | --- | --- | --- |
| 2011 | 0.0071 | 0.0451 | -0.4485 | -0.0696 | -2.1436 | -0.0994 | 0.0270 | -0.3831 |
| 2012 | -0.0010 | 0.0071 | 0.7027 | -0.0579 | 0.9631 | -0.2519 | -0.0222 | 0.1914 |
| 2013 | -0.0023 | 0.0059 | 0.3400 | 0.3088 | -1.0134 | 0.2156 | -0.0093 | -0.0221 |
| 2014 | 0.0072 | 0.0762 | 1.1980 | -0.1114 | -1.5258 | 0.0404 | 0.1352 | -0.0257 |
| 2015 | -0.0023 | -0.0105 | 0.4418 | 0.0115 | 2.1340 | -0.1732 | -0.0534 | 0.3354 |
| 2016 | -0.0020 | -0.0016 | -0.5350 | -0.1797 | 1.8509 | -0.1944 | 0.0963 | 0.1478 |
| 2017 | -0.0080 | 0.0715 | 0.2179 | -0.0852 | -0.8590 | -0.0437 | -0.0021 | -0.1012 |
| Average | -0.0002 | 0.0277 | 0.2738 | -0.0262 | -0.0848 | -0.0724 | 0.0245 |  |

^1^Year of birth cohort used as the validation population.

ADG = average daily gain at feedlot; DMI = average daily dry matter intake at feedlot; CWT = carcase weight; EMA = carcase eye muscle area; MBL = carcase marbling score; OSS = carcase ossification score; RIB = carcase subcutaneous fat depth at the ribs level.

**Supplementary Table 6**. LR Method estimates of GEBV dispersion (Disp_LR_) for feedlot and carcase traits from a 7-way cross-validation schema based on YOB cohorts

| Cohort^1^ | ADG | DMI | CWT | EMA | MSA | OSS | RIB | Average |
| --- | --- | --- | --- | --- | --- | --- | --- | --- |
| 2011 | 0.7430 | 0.9436 | 0.9326 | 0.9964 | 1.0622 | 1.0485 | 1.0413 | 0.9668 |
| 2012 | 1.0491 | 0.9271 | 1.0253 | 0.8808 | 1.1343 | 0.8861 | 0.8646 | 0.9667 |
| 2013 | 1.0314 | 1.5024 | 1.0396 | 1.1302 | 0.9991 | 0.8500 | 1.1192 | 1.0960 |
| 2014 | 0.7820 | 0.7995 | 0.8287 | 0.7821 | 0.9085 | 0.9962 | 0.9715 | 0.8669 |
| 2015 | 0.9644 | 1.6848 | 0.9829 | 0.9596 | 0.9324 | 0.7614 | 0.8962 | 1.0260 |
| 2016 | 1.1661 | 1.6037 | 1.0976 | 1.0124 | 0.9699 | 0.9773 | 0.8928 | 1.1020 |
| 2017 | 1.0620 | 0.3694 | 0.9905 | 0.8277 | 0.8764 | 0.9651 | 0.7215 | 0.8304 |
| Average | 0.9711 | 1.1187 | 0.9853 | 0.9413 | 0.9832 | 0.9264 | 0.9296 |  |

^1^Year of birth cohort used as the validation population.

ADG = average daily gain at feedlot; DMI = average daily dry matter intake at feedlot; CWT = carcase weight; EMA = carcase eye muscle area; MBL = carcase marbling score; OSS = carcase ossification score; RIB = carcase subcutaneous fat depth at the ribs level.

**Supplementary Table 7**. LR Method estimates of GEBV accuracy (ACC_LR_) for feedlot and carcase traits from a 7-way cross-validation schema based on YOB cohorts

| Cohort^1^ | ADG | DMI | CWT | EMA | MSA | OSS | RIB | Average |
| --- | --- | --- | --- | --- | --- | --- | --- | --- |
| 2011 | 0.4825 | 0.5595 | 0.6747 | 0.5677 | 0.5838 | 0.4620 | 0.4550 | 0.5407 |
| 2012 | 0.4600 | 0.4967 | 0.6627 | 0.5769 | 0.6686 | 0.4151 | 0.3952 | 0.5250 |
| 2013 | 0.5131 | 0.6607 | 0.6650 | 0.6227 | 0.6388 | 0.4043 | 0.4311 | 0.5622 |
| 2014 | 0.4223 | 0.5603 | 0.5729 | 0.5222 | 0.5568 | 0.5045 | 0.5208 | 0.5228 |
| 2015 | 0.4441 | 0.6094 | 0.5660 | 0.5525 | 0.5633 | 0.3800 | 0.4273 | 0.5061 |
| 2016 | 0.4610 | 0.6665 | 0.6412 | 0.5518 | 0.5892 | 0.4545 | 0.4180 | 0.5403 |
| 2017 | 0.5252 | 0.4053 | 0.6737 | 0.5561 | 0.5287 | 0.4862 | 0.4149 | 0.5129 |
| Average | 0.4726 | 0.5655 | 0.6366 | 0.5643 | 0.5899 | 0.4438 | 0.4375 |  |

^1^Year of birth cohort used as the validation population.
